# Cardiovascular Manifestations and Complications of Pheochromocytomas and Paragangliomas

**DOI:** 10.3390/jcm9082435

**Published:** 2020-07-30

**Authors:** Shams Y-Hassan, Henrik Falhammar

**Affiliations:** 1 Coronary Artery Disease Area, Heart and Vascular Theme, Karolinska Institutet and Karolinska University Hospital, 141 86 Stockholm, Sweden; shams.younis-hassan@sll.se; 2 Department of Endocrinology, Metabolism and Diabetes, Karolinska University Hospital, 141 86 Stockholm, Sweden; 3 Department of Molecular Medicine and Surgery, Karolinska Institutet, 171 76 Stockholm, Sweden

**Keywords:** pheochromocytoma, paraganglioma, cardiomyopathy, myocarditis, takotsubo syndrome, myocardial stunning

## Abstract

Pheochromocytomas and paragangliomas (PPGLs) are rare neuro-endocrine tumors. The catecholamine surge causes paroxysmal or chronic secondary hypertension. PPGLs may present as hypertensive- or PPGL-crisis with severe life-threatening cardiac and cerebrovascular complications. PPGLs-induced cardiac manifestations have been reported with diagnoses as PPGLs-induced electrocardiogram (ECG) changes “mimicking acute myocardial infarction”, arrhythmias, myocarditis, acute coronary syndrome, dilated cardiomyopathy, and lately as takotsubo syndrome. Critical analysis of these reports reveals that most of these cardiac manifestations have certain features in common. They have a dramatic clinical presentation and are reversible if the disease is treated with appropriate medical therapy and surgical resection of the PPGL tumor. They may have the same repolarization ECG changes irrespective of the clinical cardiac diagnosis, usually associated with mild to moderate elevations of myocardial biomarkers as troponins and normal coronary arteries. The histopathological findings are usually focal or multifocal in the form hypercontracted sarcomeres and contraction band necrosis (myofibrillar degeneration) with subsequent secondary mononuclear cell infiltration. Evidences argue the PPGL caused surge of catecholamines triggers hyperactivation of the sympathetic nervous system with cardiac sympathetic nerve terminal disruption with norepinephrine spillover causing the cardiac complications. A comprehensive review of various reported cardiovascular manifestations and complications of PPGLs are presented.

## 1. Introduction

Pheochromocytomas and paragangliomas (PPGLs) are rare neuro-endocrine tumors where the first arise from chromaffin tissues in the adrenal medulla and the second develop from chromaffin tissues in the extra-adrenal sympathetic and parasympathetic nervous system [1,2]. In 1886, Dr. Felix Frankel described, on autopsy of a patient who collapsed suddenly and died, bilateral tumors of the adrenal gland [3]. Twenty-six years later in 1912, Dr. Ludwig Picks reported and coined the term pheochromocytoma [3].

PPGLs have an annual incidence of 3–8 cases per one million per year in the general population [4]. PPGLs synthesize, store, metabolize, and usually but not always secrete catecholamines (predominantly norepinephrine) [5]. The presentation of PPGLs may be vague and the interpretation of symptoms and signs may be difficult, which may explain the delay in the diagnosis in many cases [6]. PPGLs are more frequent in patients with adrenal incidentaloma with 0.6% to 4.2% being affected [7,8,9]. Currently, most PPGLs are diagnosed due to an incidentaloma, followed by the manifestation of catecholamine excess and finally because of screening in a previously known familial syndrome [2,6,10,11]. The clinical picture of PPGLs is depending on the type and amount of the catecholamine produced and grouped accordingly [5]. Norepinephrine-mediated alpha receptor stimulation results in vasoconstriction, volume contraction and hypertension; on the other hand, epinephrine beta 2 receptor stimulation results in skeletal muscle vasodilatation and hypotension [5]. PPGLs with predominantly dopamine-secreting tumors are rare. Association between dopamine hypersecretion and more aggressive malignant disease has been observed by some investigators [5,12]. The cardiovascular manifestations, including hypertension, associated with dopamine hypersecretion is not well-studied but in purely dopamine-producing tumors these are probably uncommon. The main features of PPGLs are paroxysmal palpitation, headache, sweating, pallor, tremors, and anxiety associated with paroxysmal or sustained hypertension [6,13]. Adrenal medullary hyperplasia is a precursor of pheochromocytoma, usually found due to screening of a familial syndrome, with milder symptoms and signs [14]. There are several familial syndromes (inherited tumor syndromes) with disease-causing mutations that are associated with PPGL (e.g., *RET*, *SDHx*, *VHL*, *NF1*, *MAX* and *TMEM127*) [2]. The biochemical phenotypes associated with the different genetic causes of PPGL are slightly different. For example, *VHL*-associated pheochromocytomas are norepinephrine-secreting, whereas *SDHx* tumors may secrete dopamine and norepinephrine and *MEN2A*-related pheochromocytomas may secrete norepinephrine and epinephrine. Clinically, consideration of possible genetic causes for PPGLs determine the clinical management of such patients. For example, 123I-MIBG functional imaging is not useful in patients with *SDHx* mutations. Similarly, these patient groups require lifelong surveillance for both PPGLs and other related tumors.

The cardiovascular manifestations of PPGLs are related either to longstanding hypertension in patients with undetected PPGLs or the episodes of profuse catecholamine secretion. Severe cardiovascular complications in PPGLs are usually associated with hypertensive crisis. Different types of cardiovascular complications may occur in one fifth to more than one third of patients with PPGLs [6,15]. Zhang et al. reported on the association of pheochromocytoma and various types of cardiomyopathies [16]. They studied 163 case from 150 published articles between 1991 and 2016 (dilated cardiomyopathy *n* = 63, classical-apical takosubo syndrome (TS) *n* = 38, inverted TS *n* = 30, hypertrophic obstructive cardiomyopathy *n* = 10, myocarditis *n* = 8, and unspecified cardiomyopathy *n* = 14). There was improvement of cardiomyopathy in 96% of patients after resection of the pheochromocytoma while lack of resection resulted in either death or cardiac transplantation in 44%.

In this report, the various cardiovascular manifestations and complication induced by PPGLs are reviewed and a short description of the pathogenetic mechanism and management are presented.

## 2. PPGLs and Hypertension

PPGLs are reported in 0.1% to 0.6% of hypertensive patients [5]. Hypertension, which may be paroxysmal or chronic, is a common manifestation and may occur in up to 95% patients with PPGLs [5,6,17]. Hypertension in PPGLs is sometimes associated with symptoms as headaches, palpitation and profuse sweating. The blood pressure is usually described as fluctuating hypertension with cyclic bouts of hypertension and hypotension [5,18]. Hypertensive crisis in association with PPGLs and multisystem crisis may occur with dreaded cardiovascular and cerebrovascular complications [18,19]. Hypertrophic cardiomyopathy is one of the reported manifestations of PPGLs [20,21] due to longstanding hypertension in undetected PPGLs. Clinical and echocardiographic features may simulate that of hypertrophic obstructive cardiomyopathy [20,21], and improve or resolve after resection of the PPGL. Acute left ventricular outflow tract obstruction as a complication of PPGL-induced TS may cause systolic anterior motion of mitral valve and may be misdiagnosed as hypertrophic cardiomyopathy [22]. This type of complication is reversible after resection of the PPGL. Apical hypertrophy of the left ventricle described as “of the Japanese type” has been reported [23]. This was associated with giant T-wave inversions and was completely reversible after resection of the pheochromocytoma. After resection, 38–94% have shown improved blood pressure [24,25].

## 3. PPGL- and Hypertensive-Crisis

Pheochromocytoma-, hypertensive-, or catecholamine-crisis has been used to describe a life-threatening complication of PPGLs. This is caused by massive catecholamine surge resulting in severe labile hypertension, which may be associated with severe cardiovascular collapse, pulmonary edema and sometimes acute respiratory failure with deleterious consequences [18]. PPGL-crisis occurs usually in patients with undiagnosed PPGLs [6]. The condition may be triggered by intense psychological stress as anxiety, severe pain or exertion or occur secondary to induction of anesthesia or intubation [5]. Mechanical stress as coitus, defecation, palpation of the tumor may induce the PPGL-crisis. In pregnancy, fetal movement, excessive uterine contractions, normal parturition or cesarean section may precipitate PPGL-crisis [26]. Drugs that may precipitate a PPGL-crisis are glucocorticoids (e.g., dexamethasone, prednisone, hydrocortisone and betamethasone), dopamine receptor antagonists (including some antiemetics and antipsychotics, e.g., metoclopramide, chlorpromazine and droperidol), beta-receptor blockers, opioids (e.g., morphine, pethidine and tramadol), sympathomimetics (e.g., ephedrine, fenfluramine and dexamfetamine), norepinephrine reuptake inhibitors (including tricyclic antidepressants, e.g., amitriptyline and imipramine), serotonin reuptake inhibitors (extremely rare, e.g., paroxetine and fluoxetine), monoamine oxidase inhibitors, anticholinergic drugs (e.g., atropine), peptides (e.g., ACTH and glucagon), neuromuscular blocking agents (e.g., succinylcholine, tubocurarine and atracurium) and catecholamine–sensitizing anesthetics (e.g., halothane and desflurane) [1,19,26]. To avoid the risk of hypertensive crisis, it is important that the patient has normal plasma/urine metanephrines before a dexamethasone suppression test is performed, e.g., during the investigation of adrenal incidentaloma. Spontaneous PPGL-crisis has been observed without any exogenous stress [5,6]. Severe hypotension may be one of the features PPGL-crisis. Predominantly epinephrine-secreting tumors may present with severe hypotension since epinephrine acts mainly on beta2-adrenoceptors mediated peripheral vasodilatation [5]. PPGL-induced cardiogenic shock is usually associated with severe hypotension, which is typically caused by pump failure due to severe left ventricular dysfunction [13,27]. However, it should be remembered that severe hypotension may be caused by severe left ventricular outlet tract obstruction as a complication of mid-apical TS triggered by PPGL, a complication that is important to recognize because the treatment is completely different from cardiogenic shock caused by cardiac pump failure [27,28]. Norepinephrine-secreting tumors cause hypertension due to alpha 1-adrenosceptors mediated peripheral vasoconstriction [5]. In some cases, the circulatory and the respiratory failure are so severe that it requires treatment with extracorporeal membrane oxygenation (ECMO) to stabilize the patient’s condition until the PPGL is diagnosed and treated [18]. These life-threatening complications have been reported in a substantial number of patients with PPGLs [6,29]. In a study of 135 patients with pheochromocytomas, 15 (11%) developed acute life-threatening complications requiring treatment in the intensive care unit. The majority were of cardiac origin, including death of refractory cardiogenic shock [29]. Patients with repeated hypertensive crisis may be complicated by intracerebral bleeding [30].

## 4. PPGLs and Electrocardiogram Changes

**Figure 1:**
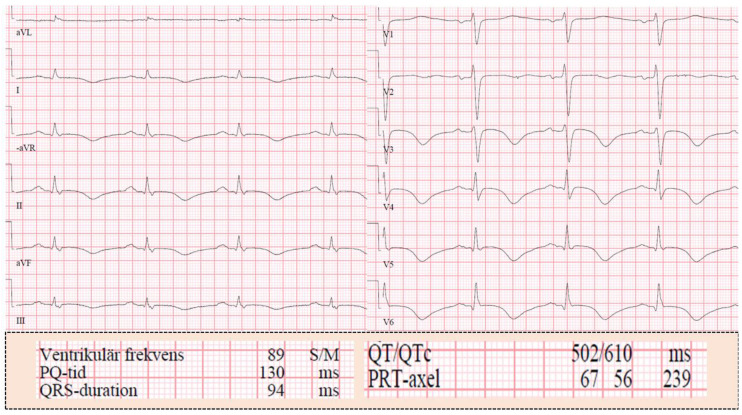
Twelve-lead electrocardiogram (ECG) in a patient with pheochromocytoma-induced mid apical takotsubo syndrome (TS). The ECG reveals general T-wave inversions with marked prolongation of the corrected QT interval 610 msec.

In the early reports on PPGLs, cardiac manifestations were reported with abnormal electrocardiogram (ECG) [31] or catecholamine-induced ECG changes mimicking ischemia induced by PPGLs [32]. Among the first ECG changes reported in patients with PPGLs were sinus tachycardia, wandering rhythm, and changes believed to be attributed to myocardial “ischemia” or “strain” in the form of diffuse ST-segment and T-wave changes [32]. During manipulation of the tumor at surgery, intermittent wandering pacemaker between the sinoatrial and atrioventricular nodes and occasional premature contractions has been observed [31]. Pheochromocytoma with ECG changes mimicking myocardial ischemia as ST-elevation or depression or T-wave inversions has been described [32]. ST-segment and T-wave ECG changes in patients with PPGLs have deemed to be due to “ischemia” and sometimes to “myocarditis” [33]. Some investigators have reported that the “myocardial infarction-like ECG changes” induced by PPGL and reverted to normal after initiation treatment with alpha receptor blocker [33] or after the tumor has been resected [34]. Others have reported giant T-waves in PPGL patients and these giant T-waves have reverted after treatment with sodium nitroprusside [35]. Giant T-wave inversions in association with left ventricular hypertrophy “of the Japanese type” caused by PPGL has been reported [23]. It was noted later that one important ECG changes is repolarization ECG changes associated with prolongation of corrected QT interval. The repolarization changes may include ST-depression or ST-elevation, peaked T-waves or giant inverted T-waves [36] (Figure 1). Interestingly, ECG changes in the form of ST-elevations and giant inverted T-waves were associated with mid-apical pattern of TS and ST-depression and peaked T-waves were associated with basal pattern of TS [37]. The peaked T-waves and the giant T-wave inversion triggered by PPGLs have been observed also to be triggered by intracranial diseases (cerebral T-waves), ischemic heart disease (Wallen’s T-waves), and TS and have been coined as sympathetic T-waves [36].

## 5. PPGLs and Arrhythmias

Different types of tachyarrhythmias have been reported in patients with PPGLs [15]. Sinus node dysfunction and brady-arrhythmias are uncommon in PPGLs [38]. Sinus pauses, and junctional escape rhythms have also been described. The sinus node dysfunction has resolved completely after PPGL removal [38]. Atrioventricular (AV) block is a rare complication. Rostoff et al. reported a symptomatic paroxysmal third-degree AV block detected during 24-h Holter monitoring, which resulted in treatment with temporary pacing [39]. The most important clinical symptom of arrhythmia in PPGLs is palpitation [6]. This may be attributed to sinus tachycardia or any other arrhythmia as supraventricular or ventricular arrhythmias. Multi-focal ventricular tachycardia has been reported [40]. Zelinka et al. reported arrhythmias in 15 (10.3%) out of 145 patients with pheochromocytoma [15]. Atrial fibrillation occurred in 9 patients (paroxysmal in 7 and permanent in 2); ventricular tachycardia in 2 where one of them was torsade de pointes. Brady-arrhythmias occurred in 3 patients (Mobitz type II second degree AV block in 1, junctional bradycardia in 2 where one of them needed pacemaker insertion). Diverse arrhythmias and life-threatening ventricular fibrillation may occur in association with surgery in patients with occult PPGL [41]. In a review of 80 patients with PPGLs-induced TS, 9 (11%) cases presented with sinus tachycardia and 2 (2.5%) cases presented with arrhythmias. A further 5 (6%) cases developed different types of arrhythmias during admission days [37]. One of the ECG changes in PPGL-induced cardiac complications is the repolarizations ECG changes in the form of peaked T-waves and the giant-wave inversions associated with prolonged corrected QT times (Figure 1). These changes may precipitate ventricular arrhythmias as torsade de pointes, which may degenerate to ventricular fibrillation [36], and potentially death.

## 6. PPGLs and Acute Coronary Syndrome

**Figure 2:**
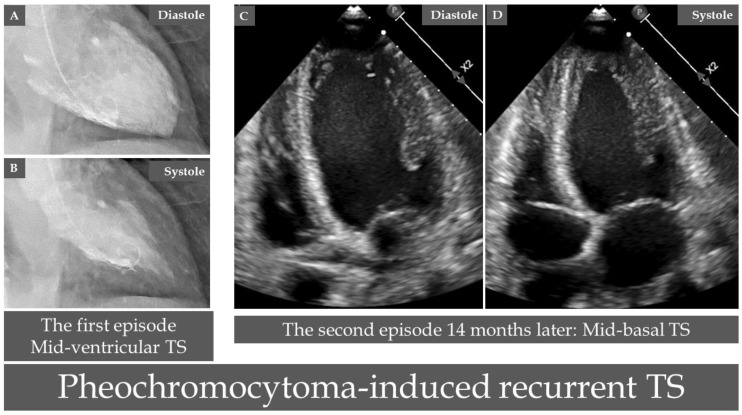
A case of recurrent takosubo syndrome (TS) induced by a pheochromocytoma. Contrast left ventriculography reveals mid-ventricular pattern of TS during the first episode ((**A**), diastole and (**B**), systole). During recurrent TS 14 months after the first episode, echocardiography reveals typical mid-basal (inverted) pattern of TS ((**C**), diastole, and (**D**), systole). Interestingly, the case was deemed as “type 2 myocardial infarction” during the first episode when pheochromocytoma was not diagnosed.

Myriads of patients with PPGLs have presented with a clinical picture of acute coronary syndrome (unstable angina pectoris, non-ST-elevation myocardial infarction (NSTEMI), and ST-elevation of myocardial infarction (STEMI)) [42,43]. Most of these patients had patent coronary arteries; some of them had “confirmed myocardial infarction” according to the authors [44]. Anterior STEMI due to thrombus occlusion of left anterior descending artery in association with pheochromocytoma has been reported [45]. In 1954, Priest et al. reported on the case of a 22-years-old farm-worker, with a “fatal myocardial infarction” [44]. The patient collapsed while fielding at a cricket match. ECG according to the authors revealed changes of “posterior infarction”. The patient died abruptly on the fifth day. Postmortem examination showed “a recent infarction of the posterior wall” and evidence of coronary plaque with a thrombus in a coronary artery in addition to a pheochromocytoma. Other investigators reported focal “myocardial necrosis” as a myocardial infarction on autopsy but the coronary arteries had minimal atheromatous changes in a patient with malignant pheochromocytoma [46]. In the early reports on PPGLs, the diagnosis of coronary insufficiency or myocardial infarction was relied in most cases on the ECG changes [42,43]. Boldt et al. reported on recurrent painless myocardial infarction based on ECG changes in a patient with a pheochromocytoma [42]. Other investigators have performed successful PPGL removal 4 weeks after myocardial infarction based on ECG changes in the form of first peaked T-waves and then extensive T-wave inversions [47]. Bourke et al. described a patient presented with pheochromocytoma-induced fatal pulmonary edema [48]. Histology of the myocardium was interpreted as “extensive myocardial damage with infarction and polymorphic cell infiltration” but the coronary arteries were normal. The authors deemed the case as metabolically induced coronary insufficiency due to demand/supply imbalance caused by intense adrenergic stimulation. Such an example is demonstrated in Figure 2 in a patient with recurrent pheochromocytoma-induced TS; the first episode was deemed by the treating cardiologists as “type 2 myocardial infarction”. A pheochromocytoma presenting with chest pain, troponin elevation and normal coronary angiography, and deemed as myocardial infarction with non-obstructive coronary arteries (MINOCA) in a 79-years-old woman has been recently reported [49]; information on cardiac image study was lacking. Several other patients with typical features of TS have been described with the diagnosis of acute myocardial infarction [50,51,52] (Table 1). “Acute anterior myocardial infarction with non-Q reinfarction” in association with a pheochromocytoma has been described in a 30-year-old pregnant woman during the 33rd week of gestation [53]. 

Stowers et al. reported on a cardiac paraganglioma compressing the left main stem coronary artery and causing angina pectoris [62]. The same authors have reviewed several other cardiac paragangliomas developing in the left atrium, interatrial septum and around coronary arteries with compression of coronary arteries. Prinzmetal angina has also been reported in patients with PPGL associated with profound hypertension [63]. Some investigators have attributed spasm angina to initiation treatment with beta blockers where intense unopposed alpha receptor stimulation can precipitate coronary artery spasm in susceptible individuals [63]. 

## 7. PPGLs and Dilated Cardiomyopathy

Dilated cardiomyopathy (DCM) has been reported as one important complication of PPGLs [64]. In early reports, the diagnosis of PPGLs-induced DCM were in most cases based on a clinical presentation with congestive heart failure, pulmonary edema or cardiogenic shock associated with diffuse left ventricular (in some cases biventricular) dysfunction documented by cardiac image studies [64,65]. DCM may occur as a complication of PPGLs with or without hypertension [64]. Compared to hypertension, present in most patients with PPGLs, PPGL-induced cardiomyopathy is relatively rare and occurs in up to 11% of the patients [66]. DCM was transient in most cases when PPGLs was detected early and treated appropriately [67] or was progressive and chronic when the tumors remained undetected [68]. Garcia and Jennings reported a case with pheochromocytoma masquerading as a cardiomyopathy [65]. The authors deemed the case as catecholamine-induced myocarditis causing cardiomyopathy; consequently, the case was diagnosed as both myocarditis and cardiomyopathy. Removal of the tumor resulted in marked improvement in the hemodynamic findings and disappearance of symptoms. End stage DCM in another case has resulted in heart transplantation because of an occult PPGL [68]. Autopsy of the diseased heart revealed “focal myocarditis” and contraction band necrosis. Interestingly, cases presenting with PPGL-induced heart failure with diffuse left ventricular dysfunction have been published using different cardiac diagnosis as PPGL-induced DCM mimicking acute coronary syndrome [69] or as fulminant myocarditis [39]. Sardesai et al. reported on 6 patients with pheochromocytomas presenting with heart failure and deemed as “catecholamine-induced cardiomyopathy” [70]. Five of the patients died of pulmonary edema within 24 h of the onset of symptoms. At autopsy, the histology in 4 of the patients showed evidence of catecholamine-induced heart disease in the form of focal myocardial necrosis with inflammatory cell response. Critical review of some cases published with cardiomyopathy reveals that they suffered of TS (Table 1) [55,57,60].

## 8. PPGLs and Myocarditis

Catecholamine myocarditis as a complication of PPGLs has been reported since decades [71,72]. The diagnosis of PPGL-induced myocarditis was based on the clinical picture, ECG changes [73], histopathological features through either endomyocardial biopsy [74] or autopsy [71], and cardiac magnetic resonance imaging (C-MRI) [61]. In 1966, Van Vliet et al. reported that 15 (58%) of 26 patients who died due to pheochromocytoma disclosed disseminated focal myocardial lesion which the authors designated “active catecholamine myocarditis” [71]. This myocarditis was characterized by focal myocardial degeneration and necrosis with foci of inflammatory cell infiltration as histocytes, plasma cells and occasional polymorphonuclear leucocytes. Jepson et al. reported on 2 deaths associated with previously unsuspected pheochromocytoma [72]. Post-mortem examination revealed “myocarditis”, which was the cause of fatal cardiac arrhythmias according to the authors.

The cardiac histopathological feature in patients with PPGLs is the focal contraction band necrosis, with inflammatory cell infiltration and fibrosis [75]. This is most probably caused by local norepinephrine spillover at the sympathetic nerve terminals [27]. Baratella et al. reported on the case of a 25-years-old woman with pheochromocytoma-induced reversible left ventricular dysfunction where endomyocardial biopsy revealed diffuse interstitial inflammatory cell infiltration with lymphomonocytes and myocardial necrosis; the patient had normal coronary arteries [74]. The patient was reported as “an unusual case of myocarditis”. Based on inflammatory cell infiltration, substantial number of patients with PPGL-induced left ventricular dysfunction have been diagnosed with myocarditis [71]. However, it is the contraction band necrosis which invokes mononuclear cell reaction and the healing process that leads to fibrosis [76]. Consequently, the inflammatory cell infiltration is a secondary response and not a primary myocarditis.

The norepinephrine spillover results in diffuse edema detected by T2-weighted on C-MRI and patchy myocardial late gadolinium enhancement. These findings are consistent with myocarditis on C-MRI [77]. On C-MRI 59% of patients with active PPGLs reveal mid-wall, subepicardial or patchy scaring. Patients with active PPGLs had also significantly higher average myocardial native T1 values on C-MRI [28,78,79,80,81]. During follow-up C-MRI analysis, the native T1 values was significantly reduced and did not return to normal [77]. With C-MRI, Ferreira et al. demonstrated impaired left ventricular function (ejection fraction < 56%) in 38% (11 out of 29) patients [77]. The peak systolic circumferential strain, and diastolic strain rate were also impaired. They had higher myocardial T1, areas consistent with myocarditis and focal fibrosis on C-MRI. The left ventricular function returned to baseline post-surgery, however, the impairment of the post systolic strain rate and the diastolic strain rate as well as some fibrosis persisted post-surgery. Rostoff et al. reported on a patient with PPGL presenting with left ventricular dysfunction and cardiogenic shock [39]. The condition was regarded as fulminant adrenergic myocarditis based on clinical, laboratory and with C-MRI, which revealed myocardial edema in the lateral, inferior and posterior wall. Performing C-MRI in patients with PPGLs, cardiac involvement will be detected frequently with changes consistent with myocarditis, focal and diffuse fibrosis, and left ventricular dysfunction [77]. These changes cannot be explained by the effects of hypertension. Cases with clear findings consistent with PPGL-induced TS have been published with the diagnosis myocarditis (Table 1) [58,61]. Based on patchy late gadolinium enhancement on C-MRI, some cases were deemed as PPGL-induced myocarditis [61]. Worth mentioning, C-MRI may show patchy late gadolinium enhancement in one third of patients with TS [80].

## 9. PPGLs and Takotsubo Syndrome

TS is currently regarded as a new acute cardiac disease entity [28,82]. The term tsubo-shaped or takotsubo-shaped were introduced by Sato and Dote in the early 1990s to describe the shape of the left ventricle during systole in patients with a clinical picture of myocardial infarction but no obstructive coronary artery disease [83,84,85]. Innumerable trigger stressors have been reported to trigger TS, among which PPGLs are well-recognized trigger factors [13,37]. The prevalence of TS in PPGLs is unknown. Giavarini et al. studied 140 patients with PPGLs and found that 15 (11%) patients suffered “acute catecholamine cardiomyopathy” [66]. Six out of 15 (or 6 (4.3%) out of 140) patients displayed classical mid-apical or inverted (mid-basal) TS. The remainder with a clinical picture of pulmonary edema had severe extensive or global left ventricular hypokinesia. This reversible global left ventricular dysfunction may be interpreted as global TS [37]. In another report, acute TS was found in 4 (2.6%) out 152 patients with PPGLs [86].

The mean age in 107 published cases with PPGLs-induced TS was 47.1 ± 15.9 years, which was about 19 years younger than other TS populations together (TS triggered by emotional stress factors, other non-PPGL acute medical or surgical disease conditions, and TS population who had no preceding trigger factors) [13]. Female patients constituted 73% of the study. STEMI-like ECG changes was found in 36.5% and ST-depression in 26%. The TS localization pattern was apical in 43.9%, mid-ventricular in 5.6%, basal in 26.2%, global in 20.6%, focal in 0.9%, and unidentified in 2.8%. PPGL-induced TS was characterized by a dramatic clinical presentation and high in-hospital complication rate occurring in 71.8%, including cardiogenic shock in almost 40% and death in 3.7%. TS recurrence rate was reported in 16.8%, which was attributed in most cases to undiagnosed PPGLs [13,27]. Left ventricular outlet tract obstruction may occur and some cases have been explained as secondary hypertrophic cardiomyopathy [22]. The condition has recovered after medical treatment or resection of the tumor [13,27].

## 10. PPGLs and Thrombo-Embolism

Left ventricular thrombus, coronary thrombosis, and peripheral embolism have been reported in patients with PPGL-induced cardiac complications [13,27,37,50,87]. Chen et al. reported on a case of 65-years-old male patient who in association with a PPGL-crisis developed acute anterior STEMI [45]. Coronary angiography confirmed thrombus occlusion of the left anterior descending artery. The patient also had left ventricular thrombus. Dagartzikas et al. reported on a 13-year-old boy with acute hemiparesis due to occlusion of M1 segment of the right middle cerebral artery causing infarct involving the right frontal and anterior temporal lobes, including the basal ganglia and insula [88]. Further investigation revealed “dilated cardiomyopathy” with left ventricular thrombus as the etiology of the cerebrovascular event.

**Figure 3:**
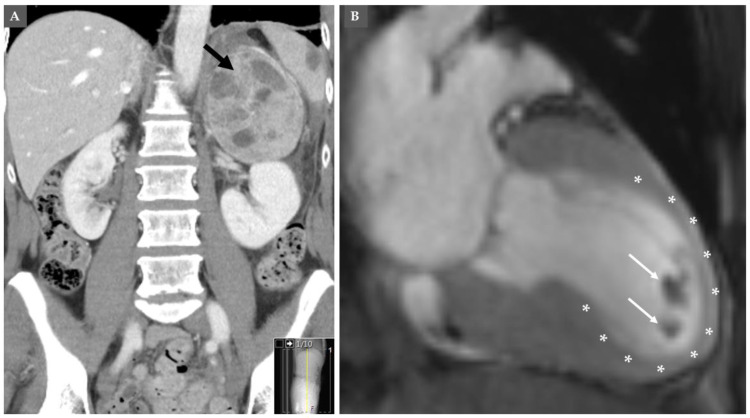
Computed tomography (CT) of the abdomen shows left-sided pheochromocytoma shown in (**A**) (black arrow). This pheochromocytoma has induced mid-apical pattern of takosubo syndrome (asterix), which was complicated by left ventricular thrombus at the apical region of the left ventricle (white arrows) as shown by cardiac magnetic resonance imaging in (**B**).

Cerebrovascular complication has been reported in 7 out of 145 (4.8%) patients with pheochromocytomas; transient ischemic attacks in 3 cases, stroke in 2 cases, subarachnoid hemorrhage in 1 case, and 1 patient suffered diffuse neurological impairment due to multiple ischemic white matter lesions [15]. Others have reported occipital cerebral infarction in pheochromocytoma-triggered mid-apical TS [89]. The most common site of left ventricular thrombus was the left ventricular apical region in mid-apical TS pattern [37,87]. In a review of 80 patients with PPGLs-induced TS, thrombo-embolic complications occurred in 6 (7.7%) [37]. Five out of 6 of thrombo-embolic complications occurred in the apical-TS pattern [37]. Left ventricular thrombus complicated by multiple cerebral emboli and bilateral renal infarction has been reported in TS triggered by pheochromocytoma [90]. A case of left ventricular thrombus in a patient with mid-apical TS triggered by pheochromocytoma is demonstrated in Figure 3.

## 11. PPGLs in Children and Cardiovascular Complications

Among PPGL tumors, 10–20% are diagnosed during childhood at an average age of 11 years with a slight predominance in boys [91]. PPGL incidence varies from 0.2 to 2 cases per million children per year. In most cases, PPGLs are sporadic in pediatric population but they may also be part of more complex hereditary syndrome. In one study constituting of 50 patients with PPGL, 56% had pheochromocytoma and 44% had paraganglioma [92]. Complication of massive catecholamine secretion can include hypertensive crisis, cardiomyopathy, pancreatitis, seizure, stroke and even multiorgan failure and death [91]. Association of “acute myocardial infarction” and pheochromocytoma has been reported in a teenage girl [93]. Acute hemiparesis due to cerebral infarction secondary to left ventricular thrombus as a complication pheochromocytoma-induced “dilated cardiomyopathy” in a 13-year-old boy has been reported [88]. Imperato-McGinley et al. reported on pheochromocytoma-induced severe dilated cardiomyopathy in a 12-year-old girl who was treated medically during seven months with the alpha-adrenergic receptor-antagonist phenoxybenzamine and tyrosine hydroxylase inhibitor alpha-methyl-para-tyrosine (MPT) to decrease her plasma catecholamine levels with moderate improvement of her left ventricular function [94]. Eighteen months after surgical removal of the tumor, there was normalization of left ventricular function. In children affected by PPGLs, genetic test should always be considered, and genetic counseling should be offered to their families.

## 12. Histopathology of Myocardium in PPGLs-Induced Cardiac Complications

**Figure 4:**
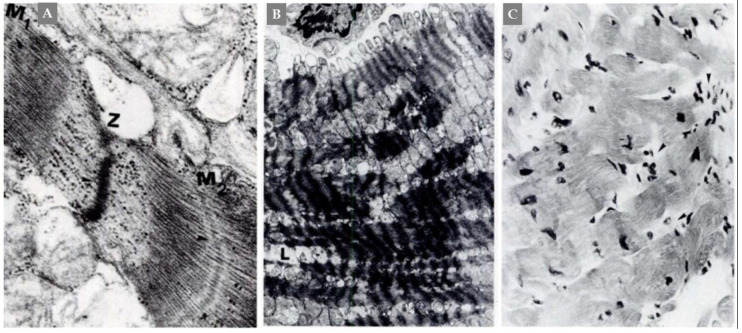
A case of catecholamine-induced “dilated cardiomyopathy”. Panel (**A**), detail of contraction bands that suggests mechanism of damage produced by supercontraction. Two hypercontracted hemisarcomeres delimited by arrows (Z-M1) and (Z-M2) are shown. Between them, initial myofibrolysis is evident. Panel (**B**), supercontraction of sarcomeres associated with intracellular Ca overload and progressing focally to myofibrolysis (L). Panel (**C**), contraction band necrosis of myocardiocytes with foci of inflammatory reaction (arrows). From Frustaci et al., Chest 1991:382-85 with permission.

The most characteristic and consistent histopathological features of PPGL-induced cardiac involvement are hypercontracted sarcomeres and contractions band necrosis (CBN) [95] (Figure 4A,B). CBN is also known as myofibrillar degeneration and sometimes referred as coagulative myocytolysis [75,96]. In CBN, there will be disruption of the linear arrangement of the myofibrils and in some areas eosinophilic transverse bands are seen to appear between zones. In severe cases, the hypercontracted sarcomeres may rupture, and the myocardium is fragmented [75]. These changes are seen early in the disease process. The myofibrillar remnants may be progressively destructed with monocyte/macrophages infiltration (Figure 4C) leading to an alveolar pattern formed by empty sarcolemmal tubes. This is followed by a healing phase with progressive collagenization and fibrosis [96]. The CBN lesions occurs in focal or multifocal pattern and apparently unrelated to blood supply and may be found in myocytes adjacent to normal capillaries. External administration of catecholamine [97], different types of intracranial processes as subarachnoid hemorrhage [98], TS even if triggered by other stressors than PPGLs [99] may cause CBN lesions. Some investigators believe that these lesions are caused by sympathetic storm resulting in disruption in cardiac sympathetic nerve terminal with norepinephrine spillover [75,100]. In patients dying suddenly due to PPGLs, the cardiac involvement may be detected by observation of CBN or myofibrillar degeneration [101].

## 13. Is There a Common Thread Tethering the Cardiac Manifestation Induced by PPGLs?

Critical analysis of the history, cardiac manifestations, ECG changes, different cardiac image studies, histopathological findings in PPGL-induced cardiovascular complications reveal that there is a common thread, which tightly unite all of them as follows:

First, irrespective of the cardiac presentation of PPGLs (as heart failure, pulmonary edema, arrhythmias, cardiac arrest, and cardiogenic shock) and the final cardiac diagnosis, which deemed to have been induced by PPGLs (as dilated cardiomyopathy, catecholamine cardiomyopathy, myocarditis, acute coronary syndrome, and TS), all these PPGL-induced cardiac manifestations have certain features in common [13,27,102]. All the cardiac manifestations have a dramatic clinical presentation with high complication rates especially if the diagnosis is delayed [13,37]. The condition is reversible if treated with appropriate medical therapy and after the PPGL tumor resection [13]. The repolarization ECG changes, mild-moderate elevation of cardiac biomarkers as troponin and usually normal coronary arteries are common findings in all of them [28,36]. The histopathological changes are usually focal or multifocal where the most dominant features are hypercontracted sarcomeres, CBN and in some cases followed by inflammatory especially mononuclear cell infiltration [75,96,103]. The patchy changes of late gadolinium enhancement in C-MRI, most probably, correspond to the focal histopathological changes seen and these patchy changes is usually interpreted as “myocarditis” changes [61].

Second, many cases with features and course typical for PPGL-induced TS, specially before recognition of the term takotsubo, have been published under various cardiac diagnoses [27] as recurrent myocardial infarction with left ventricular aneurysm complicated by left ventricular thrombus [50], “transient shock and myocardial impairment caused by pheochromocytoma crisis” [54], reversible catecholamine-induced cardiomyopathy [57,104], myocardial stunning-like phenomenon during a PPGL-crisis [105], myocarditis diagnosed by C-MRI [61] and so on (Table 1) [51,52,55,56,58,59,60]. Batisse-Lignier et al. reviewed systematically 145 published cases of pheochromocytoma-induced “acute and chronic cardiomyopathy” [106]. They classified them as “takotsubo cardiomyopathy” in 49 (33.8%) patients and “catecholamine cardiomyopathy” in 96 (66.2%) patients. Both groups of patients had similar clinical presentation. Acute pulmonary edema was more frequent in “catecholamine cardiomyopathy” whilst higher and better recovery of left ventricular function was observed in patients with TS. The authors considered that the two types of cardiomyopathies appeared to have different pathophysiological pathways. However, the two types of “cardiomyopathies” may be explained by the same pathophysiological mechanism and the difference is that the so called “catecholamine cardiomyopathy” is a more severe form of TS.

Third, pathogenesis of PPGL-induced cardiovascular complications: in addition to hypertension, several other pathogenetic mechanisms for the cardiac involvement in PPGLs have been discussed. Catecholamine-induced myocarditis is one of the mechanism discussed in causing congestive heart failure and cardiomyopathy [65]. In fact, myocarditis is a secondary process and occurs in response to contraction band necrosis with myocardial necrosis and myocarditis is improperly used as a primary diagnosis [76]. Inadequate coronary perfusion caused by increased myocardial demand (supply/demand imbalance) is another discussed mechanism [48]. The histopathological finding of hypercontracted sarcomeres and CBN with secondary mononuclear cell infiltration argues strongly against myocardial infarction caused by increased myocardial demand or any other cause of ischemia as microvascular dysfunction [103]. Another discussed mechanism is the cardiac overstimulation by epinephrine leading to switch in intracellular signal trafficking from Gs (stimulation) protein to Gi (inhibitory) intracellular activation of beta 2 adrenoreceptors predominantly in the left ventricular apex resulting in decreased myocardial contractility [107]. However, the hypercontracted sarcomere and contraction in PPGL-induced myocardial stunning argues strongly against the hypothesis of epinephrine-induced switch in signal trafficking [75]. Furthermore, several other findings challenging the epinephrine hypothesis have been discussed elsewhere [108,109]. Evidence for the involvement of sympathetic nervous system and cardiac sympathetic nerve terminal disruption with norepinephrine spillover is immense [13,27]. PPGLs may be localized by meta-(123) iodobenzylguanidine (I-123-MIBG) scintigraphy, which also can be used to image the heart. Patients with PPGLs have shown inverse relationship between heart intensity in the 24-h and 48-h images of I-123-MIBG and plasma/urine catecholamine concentrations [110]. Nakajo et al. have also demonstrated rapid clearance of I-123-MIBG from the heart in patients with pheochromocytoma [111]. The myocardial retention of MIBG gradually improved after surgical resection of the pheochromocytoma. Similar findings have also reported by others [112,113]. The above findings agues for the evidence of cardiac sympathetic denervation in PPGLs. Furthermore critical review of the pathogenesis of TS [28,81] including PPGL-induced TS [27], repolarization ECG changes [36] and contraction band necrosis [75,103] argues for the involvement of the sympathetic nervous system including cardiac sympathetic disruption in the pathogenesis of PPGL-induced cardiac complications [36,103,114,115].

Consequently, it will be justifiable to conclude that the miscellaneous cardiac manifestations are most probably tied with a common thread that is the sympathetic nervous system and the cardiac sympathetic nerve terminals disruption with norepinephrine spillover [27] which can explain both the multifocal histopathological changes seen in the myocardium in PPGL-induced cardiac manifestation and the patchy changes seen in C-MRI [27,116,117].

## 14. PPGL Management

Early diagnosis of PPGL is crucial to avoid life-threatening complications. The initial investigation for suspected cases of PPGLs includes the measurement of plasma free metanephrines and/or urinary fractionated metanephrines [1]. Computed tomography scan and MRI are valuable for initial localization of the PPGLs tumors. Functional imaging with radiopharmaceutical agents as 123-I-MIBG scintigraphy or PET (e.g., Ga-DOTATOC) for confirmation and planning correct therapy.

Surgical resection of the tumor is the only curative option [1,118]. Preoperative management consists of alpha blockade and correction of intravascular volume [5,24,27]. The medical treatment before surgery is almost entirely based on expert opinion and observational studies. Treatment with alpha adrenergic receptor blocker is considered as the treatment of choice [118]. The two most commonly used drugs are phenoxybenzamine, which is a nonselective and noncompetitive alpha-1 and alpha-2 adrenergic receptor blocker. The second one is doxazosin, which is a selective and competitive alpha-1 adrenergic receptor blocker. It is recommended to initiate one of these two drugs 1-2 weeks before surgery with increasing dosages until the blood pressure targets are achieved. In a study comparing pretreatment with doxazosin and phenoxybenzamine during PPGL surgery, there was no difference in the duration of blood pressure outside the target range during resection of PPGL-tumor. However, phenoxybenzamine was more effective in preventing intraoperative systolic blood pressure above the target range and hemodynamic instability [119]. In an acute situation, PPGL-crisis alpha-blockers in the form of intravenous phentolamine is preferred over phenoxybenzamine because it has a rapid action. Calcium channel blockers (nifedipine or amlodipine) have also been used and preferably added to alpha adrenergic receptor blocker for further improvement of blood pressure control [118]. Beta- blockers are indicated to control tachycardia. Beta- blockers should not be initiated before alpha-blockers in the setting of PPGLs because of challenges in the cardiovascular management due to unopposed alpha blockade [5]. Beta- blockers in the form propranolol, metoprolol or atenolol can be used. Evidence to support the preference of beta-1 selective blockers over non-selective beta- blockers does not exist [1,118]. Labetalol with its more potent beta- than alpha-antagonist activities is not recommended as the initial therapy because it can lead to paradoxical hypertension and even hypertensive crisis [1].

Worth mentioning is that a multi-endocrine crisis may occur as thyrotoxicosis crisis together with pheochromocytoma multisystem crises have been reported [120] and this requires intensive cardiovascular management. In such cases, alpha- and beta-blockers may be started simultaneously. Such simultaneous treatment with alpha- and beta-blockers should be considered in PPGLs-induced left ventricular outlet tract obstruction where treatment with beta-blockers is decisive. In preoperative assessment, it is mandatory to monitor heart rate, arterial blood pressure, and arrhythmias and to restore the blood volume to normal [26].

Careful follow-up to detect recurrence and prevent life-threatening complications is imperative. Different cardiovascular complications are treated accordingly. This has been discussed in detail elsewhere [5,27,28,121]. One very important point in the treatment of PPGL-induced cardiogenic shock is that inotropic medications are contraindicated [28,82,121]. Treatment with mechanical circulatory support as extracorporeal life support in cases of refractory hypotension is lifesaving [13,27].

## 15. Conclusions

PPGLs are rare neuro-endocrine tumors which may produce massive catecholamine levels causing paroxysmal or chronic secondary hypertension. PPGLs may present with hypertensive- or PPGL-crisis with severe life-threatening cardiac and cerebrovascular complications. However, the disease is curable if detected early and the tumor resected with appropriate presurgical medical therapy. During the last 70 years, the cardiac complications have been mainly published as PPGL-induced “acute coronary syndrome”, “myocarditis”, “dilated cardiomyopathy” and “takotsubo syndrome”. Critical analysis of these reports reveals that most of these cardiac manifestations have certain features in common as a dramatic clinical presentation and reversibility of the disease, repolarization ECG changes irrespective of the clinical cardiac diagnosis, usually associated with mild to moderate elevations of myocardial biomarkers as troponins and usually normal coronary arteries. The focal or multifocal histopathological changes are mainly in the form of hypercontracted sarcomeres and contraction band necrosis. This indicates that the different PPGL-induced cardiac manifestations are tied with a common thread, namely catecholamine-triggered hyperactivation of the sympathetic nervous system with cardiac sympathetic nerve terminal disruption with norepinephrine spillover.

## Figures and Tables

**Table 1:** Published cases diagnosed with various pheochromocytomas and paragangliomas (PPGL)-induced cardiac complications but had features consistent with takotsubo syndrome.

Authors and the Year of Publication	Age Gender	Published with Diagnosis in Addition to PPGL	Important other Findings and Complications	Findings Consistent with Takotsubo Syndrome
McGonigle et al., 1983 [50]	38/F	Recurrent myocardial infarction	Marked reversible ST-elevation and “left ventricular aneurysm” complicated by left ventricular thrombus	Recurrent TS
Shaw et al., 1987 [54]	41/M	Transient shock and myocardial impairment	A left ventricular angiogram showed hyperkinesis of the basal segments and the remaining areas of myocardium were akinetic	Mid-apical TS pattern
Case records of the MGH (case 15-1988) [55]	26/F	Embolic stroke of cardiac origin, cardiomyopathy	Echo: the left ventricle was markedly hypokinetic at the base and the midventricular level, and function at the apex was hyperkinetic Endomyocardial biopsy: vacuolization of the myocytes, myocyte necrosis, and scattered myocytes were hypereosinophilic with focal disruption of their sarcolemmal membrane The left ventricular dysfunction resolved completely	Mid-basal TS (inverted TS) complicated with embolic stroke
Iga et al., 1989 [56]	44/F	Reversible left ventricular wall motion impairment	A figure in the article shows an echo finding typical for mid-apical ballooning	Mid-apical TS
Elian et al., 1993 [57]	41/M	Reversible cardiomyopathy with acute pulmonary edema	Left ventricular thrombus and heart failure	Mid-apical TS
Boulmier et al., 2000 [51]	56/F	Myocardial pseudo-infarction Acute cardiomyopathy or coronary spasm	Diagnosis of myocardial necrosis, acute myocarditis	Mid-apical TS
Mauser et al., 2001 [52]	41/F	A large anterolateral-apical-diaphragmal myocardial infarction	Complicated by acute pulmonary edema and cardiogenic shock, the left ventricular dysfunction resolved after 4 weeks	Mid-apical TS
Dinckal et al., 2003 [58]	44/F	Myocarditis mimicking myocardial infarction	Echo showed basal and midventricular hypokinesia. The ECG and echo changes normalized within few days	Typical mid basal (inverted) TS
Roubille et al., 2010 [59]	35/F	Recurrent severe acute apical sparing left ventricular dysfunction	Echo reveals typical severe basal and midventricular left ventricular dysfunction but preserved apical contractility	Recurrent inverted TS
Miura et al., 2017 [60]	63/F	Reversible cardiomyopathy	Defects of MIBG uptake in the mid-apical segments corresponding to the akinetic segments with normal MIBG uptake in the normo-kinetic basal segments, and histopathological findings consistent with TS	Biventricular mid-apical ballooning pattern consistent with TS
Khattak et al., 2018 [61]	25/M	Acute myocarditis	ECG findings of wide-spread ST-depression besides echo and C-MRI findings of hypokinesis at the basal segments argue strongly for basal TS pattern (inverted TS) with late gadolinium enhancement at the basal segments	Mid-basal (inverted TS)

F, female. M, male. TS, takotsubo syndrome. ECG, electrocardiogram. MIBG, metaiodobenzylguanidine. C-MRI, cardiac magnetic resonance imaging.
